# Mitigation of liquid–liquid phase separation of a monoclonal antibody by mutations of negative charges on the Fab surface

**DOI:** 10.1371/journal.pone.0240673

**Published:** 2020-10-30

**Authors:** Tatsuji Matsuoka, Ryuki Miyauchi, Nobumi Nagaoka, Jun Hasegawa

**Affiliations:** Modality Research Laboratories, Daiichi Sankyo, Co., Ltd., Shinagawa, Tokyo, Japan; CIC bioGUNE, SPAIN

## Abstract

Some monoclonal antibodies undergo liquid–liquid phase separation owing to self-attractive associations involving electrostatic and other soft interactions, thereby rendering monoclonal antibodies unsuitable as therapeutics. To mitigate the phase separation, formulation optimization is often performed. However, this is sometimes unsuccessful because of the limited time for the development of therapeutic antibodies. Thus, protein mutations with appropriate design are required. In this report, we describe a case study involving the design of mutants of negatively charged surface residues to reduce liquid–liquid phase separation propensity. Physicochemical analysis of the resulting mutants demonstrated the mutual correlation between the sign of second virial coefficient *B*_2_, the Fab dipole moment, and the reduction of liquid–liquid phase separation propensity. Moreover, both the magnitude and direction of the dipole moment appeared to be essential for liquid–liquid phase separation propensity, where electrostatic interaction was the dominant mechanism. These findings could contribute to a better design of mutants with reduced liquid–liquid phase separation propensity and improved drug-like biophysical properties.

## Introduction

The number and variety of monoclonal antibodies (mAbs) approved for clinical trials and commercial release have been increasing continuously in recent decades [1–3]. However, unlike small-molecule drugs, manufacturing mAbs is challenging because their substantial complexity and labile conformational stability induce disorder in their higher-order structures. MAbs can be exposed to various stresses during their manufacturing processes, formulation, storage, delivery, and administration, which can result in undesirable fragmentation, aggregation, denaturation, and chemical modifications of the mAbs [4–6]. These alterations can affect the manufacturability as well as the efficacy and safety of therapeutic mAbs.

Therefore, all mAb drug candidates should undergo characterization of their physicochemical properties in order to mitigate the risks of significant instability, and this should be performed at the front end of the development process or during a discovery phase to reduce drug development costs [7, 8]. One of the key risks is the loss of colloidal stability, specifically undesirable liquid–liquid phase separation (LLPS) and increased viscosity in highly concentrated solutions [9–12]. The LLPS of mAbs could result in a poor appearance and inconsistent vial-to-vial concentrations [9].

The effect of chemical additives on formulation is systematically organized and well understood [13]. In fact, several formulation studies have reported mitigation of the LLPS propensity of mAbs [9, 14–18]. For example, adding sucrose or hydroxypropyl beta cyclodextrin [15], adding arginine glutamate [17], or adding salt or increasing the temperature mainly by decreasing protein–protein self-attractive interactions [18] mitigates the LLPS propensity of mAbs. Formulation studies by Reiche et al. [9] also showed that the overall hydrophobicity and charge distribution of proteins play a role in LLPS of mAbs. However, in our experience, such formulation studies for protein therapeutic candidates do not always work well within the limited time typically given by pharmaceutical companies for drug development.

Instead, the optimization of mAb sequence through protein engineering technology is an attractive approach for mitigating the LLPS propensity of mAbs because the molecular optimization could dramatically change it. Engineering can also be implemented in the early phase of drug development using very small amounts of mAbs. The effective *in silico* design of mAbs has previously been reported to reduce the risk of viscosity escalation [19–23]. In terms of LLPS, Du et al [24] successfully used *in silico* homology modelling and charged patch analysis to identify seven amino acids that were potentially related to self-association. They confirmed that mutations of the amino acids resulted in a lower LLPS propensity. Chow et al. [25] indicated that the disruption of the charged surface patch of an immunoglobulin (Ig) G4 suppressed its LLPS propensity. In their study, the variable region (Fv) dipole moment and the diffusion interaction parameter *k*_D_ were qualitatively correlated with the LLPS propensity of a wild type (WT) and its four mutant mAbs.

In this study, we performed protein engineering to reduce the LLPS propensity of a humanized IgG1, mAb1, and showing strong LLPS propensity. mAb1 mutants were designed with the goal of eliminating the negatively charged surface patch. The physicochemical properties of the resultant clones were characterized, and their structures were analysed using homology models. The findings revealed that both the magnitude and direction of the antigen-binding fragment (Fab) dipole moment were essential contributors to LLPS propensity.

## Materials and methods

### MAb production

Wild type (WT) mAb1 and its mutants M1–M9 were produced by HEK293 cells and purified by successive two-step chromatography [26] with MAbSelect SuRe affinity resin (GE Healthcare, Chicago, IL, USA) and ceramic hydroxyapatite type I resin (Bio-Rad Laboratories, Hercules, CA, USA). The mAb concentrations were determined by size exclusion-high performance liquid chromatography (SE-HPLC) with absorbance detection at 280 nm. Molar extinction coefficients were calculated using Sednterp software version 1.09 (National Institutes of Health, Bethesda, MD, USA). Sodium chloride was purchased from Wako Pure Chemical Industries, Ltd. (Osaka, Japan), and sodium phosphate and sodium acetate were obtained from Kanto Chemical Co., Inc. (Tokyo, Japan).

### Solution preparation

We dialyzed mAbs against buffer A (25 mM sodium phosphate/15 mM NaCl/pH 6.0) or buffer B (25 mM sodium phosphate/150 mM NaCl/pH 6.0). We used mAbs in buffer A for all measurements and used its pH and ionic strength as parameters for *in silico* analyses, as described in the Analysis of full IgG and Fab model structures subsection. We also used mAbs in buffer B for dynamic light scattering measurements.

### Analysis of full IgG and Fab model structures

Model structures of the full-length and antigen-binding fragment (Fab) of the WT mAb1 and its mutants were generated using the MODELLER implementation in Discovery studio 2017R2 software [27] (Dassault Systemes, Vélizy-Villacoublay, France) using crystal structures of an antibody (PDB ID, 1HZH) or a Fab (PDB ID, 5I19) as the template, respectively. The electrostatic potential of the surfaces was determined according to the Poisson–Boltzmann equation at pH 6.0 at an ionic strength of 43 mM, which corresponds to the ionic strength of buffer A (25 mM sodium phosphate/15 mM NaCl/pH 6.0) used for *in vitro* experiments. For force field assignment, the charge and bondi radii were applied as parameters. The +1 and −1 kBT/e isovalue surfaces were mapped onto the model structures, where kB is the Boltzmann constant. The angle *θ* (degree) of the Fab dipole moment between the WT and each mutant (Mi) was calculated with the component (Dipole *X*, *Y*, or *Z*) of each Fab dipole moment (S1 Table) according to Eq (1):

$$\theta =\frac{\pi }{180}*\cos^{-1}\left(\frac{WT\_DipoleX*Mi\_DipoleX+WT\_DipoleY*Mi\_DipoleY+WT\_DipoleZ*Mi\_DipoleZ}{\sqrt[{2}]{{WT\_DipoleX}^{2}+{WT\_DipoleY}^{2}+{WT\_DipoleZ}^{2}}*\sqrt[{2}]{{Mi\_DipoleX}^{2}+{Mi\_DipoleY}^{2}+{Mi\_DipoleZ}^{2}}}\right)$$ (1)

### Isoelectric focusing (IEF)

Samples were electrophoresed on PhastGel IEF gels (GE Healthcare Life Sciences) using the PhastSystem electrophoresis workstation (GE Healthcare Life Sciences). An Isoelectric Focusing Calibration Kit High Range pI (pH 5–10.5) (GE Healthcare Life Sciences) was used for marker proteins. The gels were stained with PhastGel Blue R (GE Healthcare Life Sciences). The experiment was performed in one replicate.

### Differential scanning calorimetry

Differential scanning calorimetry (DSC) studies were conducted using a VP capillary DSC system (Malvern Panalytical, Malvern, UK). Samples (0.5 mg/mL in buffer A) were heated from 20°C to 100°C at a scanning rate of 200°C/h. For each sample, a relevant buffer blank was subtracted using Origin 7.0 software (OriginLab Corporation, Northampton, MA, USA). The apparent *T*_m_ was determined from the exothermic peak that provided the highest heat capacity (C_P_.) The experiment was performed in one replicate.

### Phase separation experiments

The WT and mutants M1–M9 were concentrated to approximately 80 mg/mL and dialyzed against buffer A using an Xpress Micro Dialyzer MD 100 12–14 kDa cartridge (Scienova GmbH, Jena, Germany). The concentration of each dialysate was adjusted to 50 mg/mL by adding buffer A. The resulting solutions were transferred to 200-μL polymerase chain reaction (PCR) tubes (MicroAmp 8-cap strip; Thermo Fisher Scientific, Waltham, MA, USA) and stored at 4°C for 1–4 days to allow LLPS to occur. The samples in PCR tubes were mixed by gentle inversion, the temperature was raised from 4°C to 10°C, and the samples were stored overnight at 10°C. We repeated the 4°C LLPS experiments for M8 and M9 at a larger scale of 600 μL in 1.5-mL clear tubes, with WT as a reference. The tubes were incubated overnight at 4°C and then centrifuged for 48 h at 21,600 ×*g* at 4°C. Because of limited sample availability, the experiment was performed in one replicate.

### Viscosity measurement

The mAbs in buffer A were concentrated to 130–140 mg/mL using a Vivapore 5 Static Concentrator (Sartorius Stedim Biotech GmbH, Göttingen, Germany), and the concentration was adjusted to 120 mg/mL with buffer A. The viscosity of the mAb solution was measured at 25°C using an m-VROC micro-viscometer (RheoSense Inc., San Ramon, CA, USA). Because of limited sample availability, all measurements were performed in one replicate.

### Analytical ultracentrifugation-sedimentation equilibrium

Other than the following, all procedures were performed exactly as described in previous studies [28, 29]. For analytical ultracentrifugation-sedimentation equilibrium experiments, 97 μL of each mAb solution (1, 5, and 10 mg/mL) in buffer A was centrifuged in a Proteome XL-I instrument (Beckman Coulter, Inc., Brea, CA, USA). The partial specific volumes were calculated as 0.7291 (M1), 0.7291 (M2), 0.7291 (M3), 0.7293 (M4), 0.7293 (M5), 0.7293 (M6), 0.7295 (M7), 0.7292 (M8), 0.7292 (M9), and 0.7289 (WT), cm^3^/g using Sednterp software. We determined apparent molecular weights by nonlinear least-squares fitting using Origin software (OriginLab Corporation, Northampton, MA, USA). *B*_2_ was obtained from the slope of the plot between the inverse of the apparent molecular weight and protein concentration, as shown by Saito et al. [29, 30]. Because of limited sample availability, the experiment was performed in one replicate.

### Dynamic light scattering

The mAb samples at concentrations of 2.5, 5.0, 7.5, and 10.0 mg/mL (after filtration through a 0.22-μm filter) in buffer A or buffer B (25 mM sodium phosphate/150 mM NaCl/pH 6.0) were plated into wells of 384-well optically transparent-bottom microtiter plates. The diffusion coefficient *D*_s_ was measured using a DynaPro Plate Reader version II (Wyatt Technology Corp., Santa Barbara, CA, USA) at 20°C. The diffusion interaction parameter *k*_D_ was calculated using the protocol described in a previous paper [29]. The experiment was performed in duplicate.

### PEG-mediated relative solubility

Polyethylene glycol (PEG)-6000 solutions (0.5%–18.5% w/v) at 2% increments in buffer A were prepared from 50% concentrated PEG-6000 stock solutions. The mAb samples were diluted to 1 mg/mL in buffer A, and 12 μL of the mAb samples and 28 μL of PEG solution were mixed, followed by filtration 20 min later. The absorbance at 280 nm was then measured. The PEG_m_, i.e., the weight% of PEG in solution required to reduce the protein concentration by 50%, was calculated as the relative solubility [31]. The experiment was performed in duplicate.

## Results

### Physicochemical analysis of WT mAb1

The WT mAb1 underwent LLPS at 50 mg/mL at 4°C in buffer A and a relatively high viscosity of 15.43 mPa**·**s at a concentration of 120 mg/mL (Table 1). The *B*_2_ was −4.08 × 10^−5^ mL·mol/g^2^ and the *k*_D_ was −33 mL/g for the WT. The sign for *B*_2_ suggests the existence of attractive protein–protein intermolecular interactions [11, 20, 21, 26, 29, 30]. The addition of 150 mM NaCl increased the *k*_D_ value from −33 mL/g to −12 mL/g, indicating that the protein–protein self–attractive interactions in low–ionic–strength buffer A could essentially be mediated by electrostatic interactions.

**Table 1:** Biophysical assessments using *in silico* and *in vitro* analysis of wild type (WT) and mutant (M) mAbs.

Name	Mutations	Magnitude of Fab dipole moment (debye)	Angle of Fab dipole moment (°)	LLPS at (4°C)	*B*_2_ (×10^−5^ mL·mol/g^2^)	*k*_D_ (mL/g) in buffer A	*k*_D_ (mL/g) in buffer B	*PEG*m (%)	Viscosity (mPa·s)	Observed pI
**WT**		933.0	-	Detected	−4.08	−33	−12	5.62	15.43	8.17
**M1**	LC: E27A	939.7	1.84	Detected	−2.23	−22	−12	7.62	13.17	8.38
**M2**	LC: D28A	848.2	3.03	Detected	−2.62	−25	−14	7.49	11.93	8.44
**M3**	LC: D93A	886.9	0.12	Detected	−1.22	−20	−11	7.67	9.08	8.39
**M4**	LC: E27A, D28A	776.9	9.50	Not detected	0.84	−19	−11	8.25	10.57	8.50
**M5**	LC: E27A, D93A	760.1	9.30	Not detected	2.04	−14	−14	8.44	8.19	8.50
**M6**	LC: D28A, D93A	715.1	5.65	Not detected	1.88	−15	−12	8.63	8.77	8.56
**M7**	LC: E27A. D28A. D93A	674.1	10.07	Not detected	3.60	−12	−12	9.13	9.49	8.68
**M8**	HC: D31A	940.6	16.05	Opaque	−3.70	−32	−19	6.97	13.6	8.40
**M9**	LC: D56A	969.8	7.08	Opaque	−2.60	−23	−12	7.34	9.6	8.39

### Mutant design

**Fig 1:**
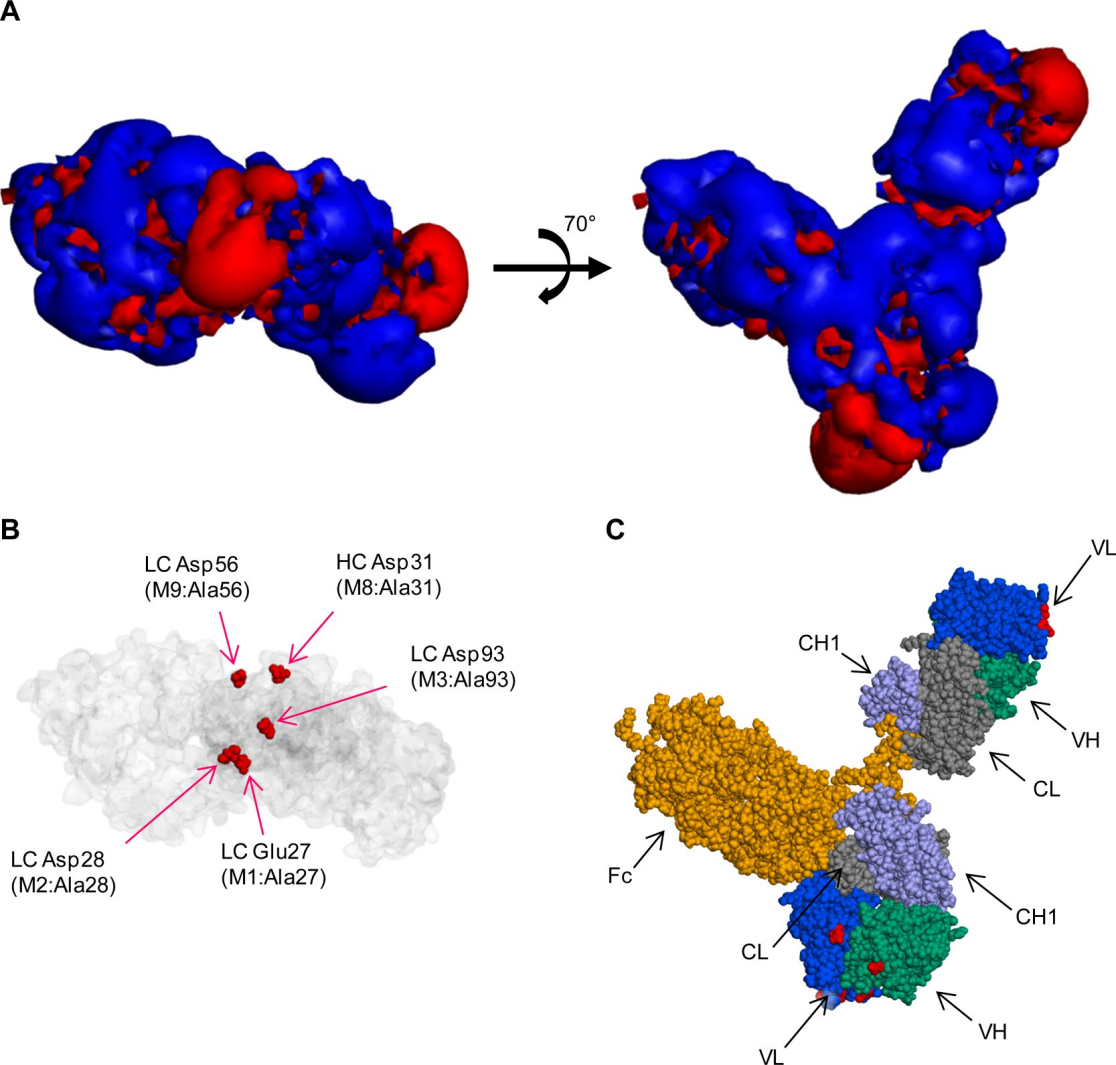
Charge distribution on the surface of WT mAb1. Three representations of full-length IgG were used. (A) Rotational view of mAb1 isovalue surface. The red and blue contours indicate the −1 *k*_B_*T*/*e* (negative) and +1 *k*_B_*T*/*e* (positive) isosurface potentials. (B). Connolly surface (transparent gray color) with Corey–Pauling–Koltun (CPK) representation (red color) of five negatively charged amino acids: light chain (LC) Glu27, LC Asp28, LC Asp93, LC Asp56, and heavy chain (HC) Asp31. (C) CPK representation of the WT, with yellow = Fc, blue = VL, green = VH, light purple = CH1, gray = CL, and red = mutated residues on Fv regions.

The relationship between the surface charge distribution and the location of negatively charged surface residues was demonstrated on a full-length IgG model of WT mAb1 (Fig 1). The negative charges on the surface of the WT mAb1 were unevenly distributed in the surface electrostatic potential maps (Fig 1A), which was previously believed to induce protein self-interactions and high viscosity [19, 20]. As shown in Fig 1C, mAb has two Fabs and an Fc; the Fab is composed of a constant domain (CL and CH1) and an Fv domain (VL and VH). Major negative surface charges were located in both Fv domains of the full-length model. They primarily arose from five acidic amino acid residues: light chain (LC) Glu27, Asp28, Asp56, Asp93, and heavy chain (HC) Asp31. These five negatively charged amino acids were highlighted on the Fv domain facing forward in Fig 1B, while not highlighted on another Fv domain located on the right side. Their positions corresponded to positions of the major negative charge patches in panel A, left. Of these, LC Glu27, Asp28, and Asp93 formed a major continuous patch of negative charges on the molecular surface.

We designed combinations of Ala-substituted mutants at the LC amino acids Glu27, Asp28, and Asp93 to disrupt the continuous negative charge patch. Specific designs were single-charge deletion mutants (M1, M2, and M3), double-charge deletion mutants (M4, M5, and M6), and a triple-charge deletion mutant (M7). In addition to these mutants, Ala-substituted mutants for HC Asp31 (M8) and LC Asp56 (M9) were designed.

**Fig 2:**
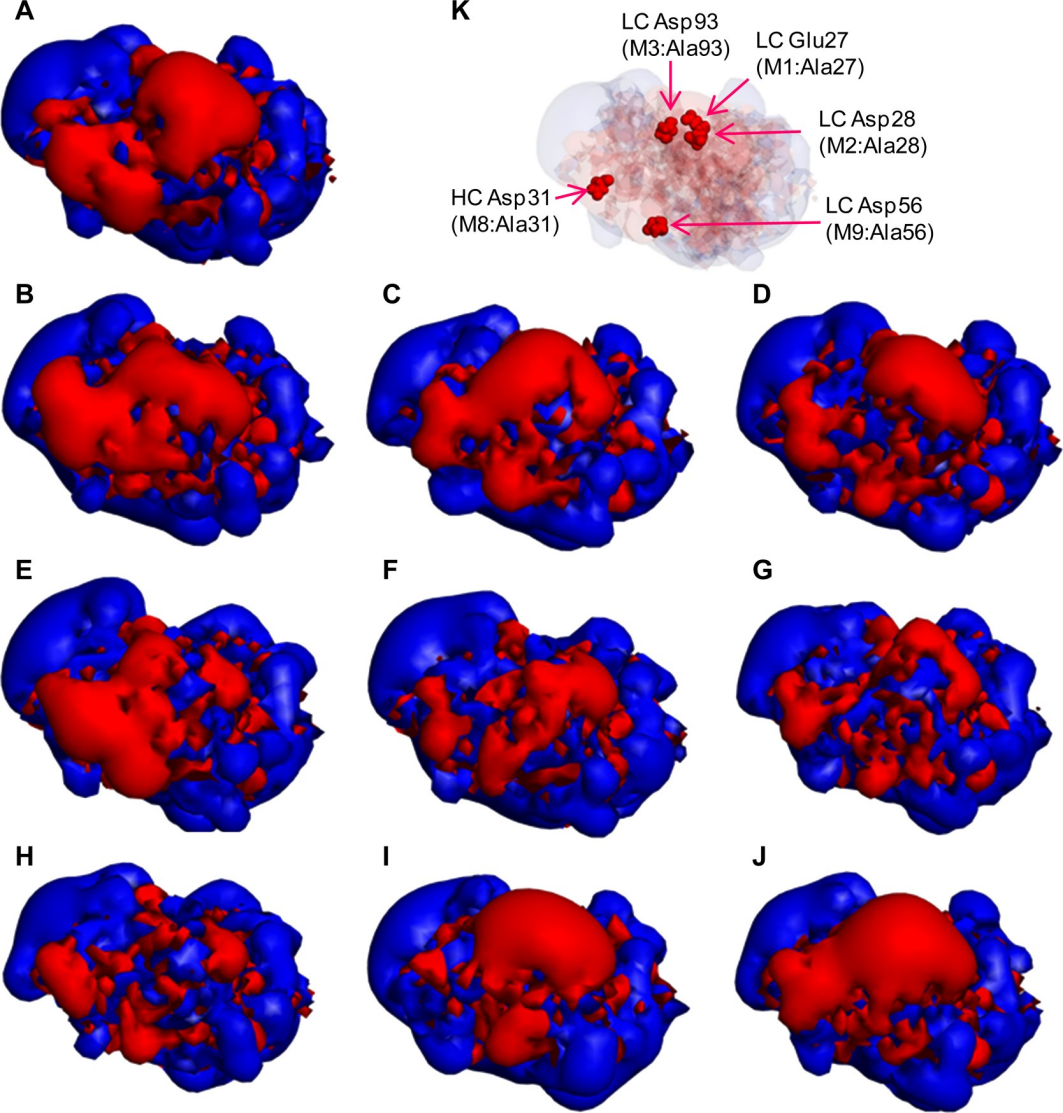
Electrostatic potential surfaces of Fabs of WT and mutants. (A) WT, (B) M1, (C) M2, (D) M3, (E) M4, (F) M5, (G) M6, (H) M7, (I) M8, and (J) M9: The red and blue contours indicate the −1 and +1 *k*_B_T/e isosurface potentials. (K) Connolly surface (transparent gray color) with a CPK model (red color) of five negatively charged amino acids: light chain (LC) Glu27, LC Asp28, LC Asp93, LC Asp56, and heavy chain (HC) Asp31.

The surface electrostatic potential of the designed Fabs are presented in Fig 2. The apparent sizes of the negatively charged patches were reduced in M1–M7 in proportion to the number of depleted negative charges. In M8 and M9, the apparent sizes of rather small negative charge patches were reduced, while the continuous patch was not reduced, as designed.

S1 Fig shows Fab dipole moments. The magnitude of Fab dipole moment was 933 debye for WT, 940–969 debye for single-charge deletion mutants, 715–777 for double-charge deletion mutants, and 674 debye for the triple-charge deletion mutant (Table 1). The angle between the WT and mutant dipole moments for M8 and M9 was larger compared with that for other single-charge deletion mutants: M1, M2, and M3.

### Physicochemical analysis of mutants

IEF was performed to confirm the charge deletions of the mutants (Table 1). The isoelectric point (pI) of each mAb was distributed from 8.17 for the WT to 8.68 for M7. The pI values correlated well with the number of charge deletions as designed.

In addition to an alterlation in charges, mutations can often cause drastic changes in the protein structure [32], which might affect LLPS propensity, viscosity, and protein solubility. The structural identities were verified using DSC. Overall, DSC thermograms of the WT and mutants appeared almost identical and had a main peak *T*_m_ of ~80°C (S2 Fig), indicating that mutations do not likely affect the conformational stability of proteins.

**Fig 3:**
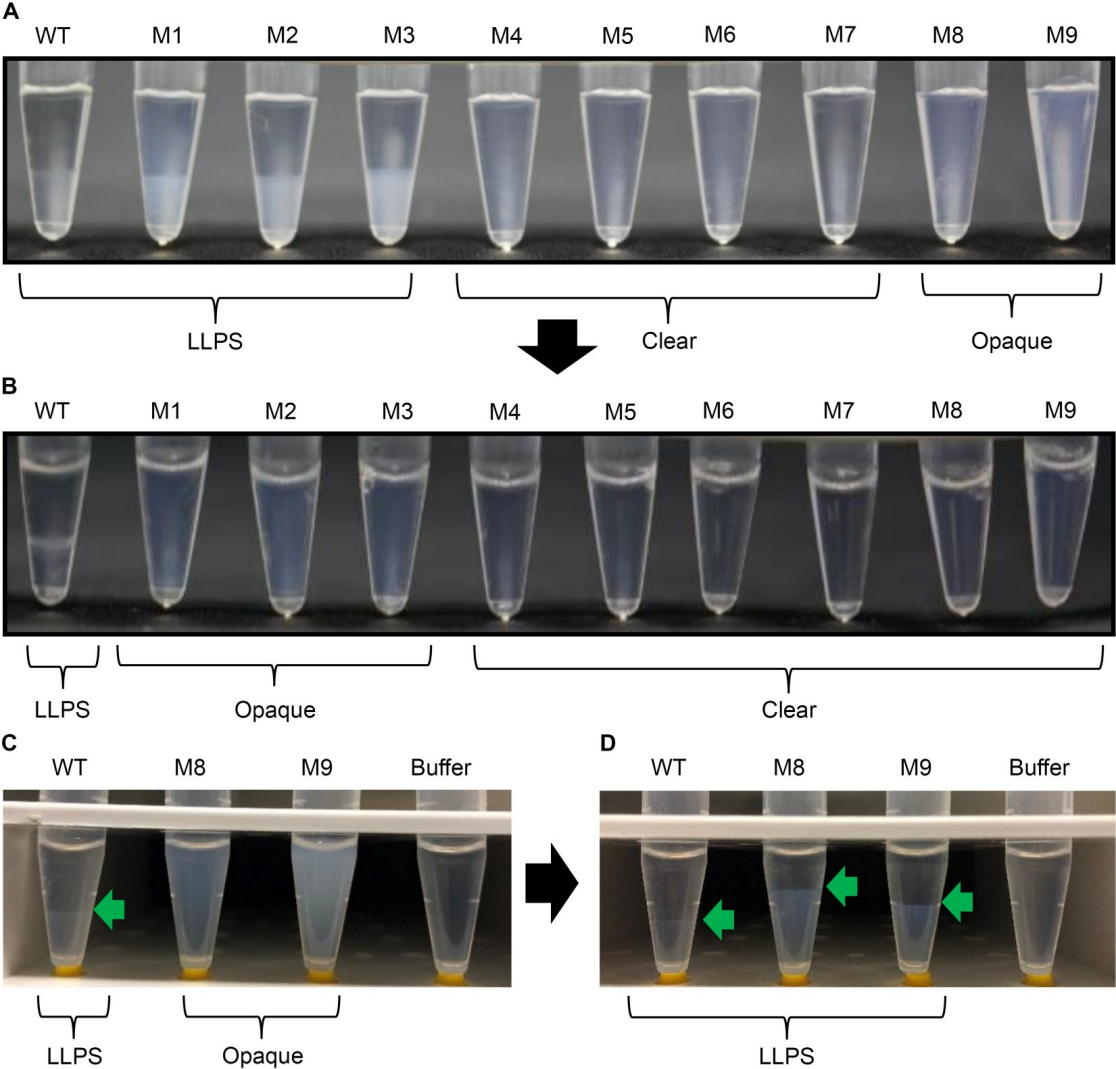
LLPS of WT and mutants at 4°C. (A) We added 100 μL of WT and mutant antibodies in 200-μL PCR tubes and stored them overnight at 4°C. (B) The samples were then mixed by gentle inversion at 4°C and stored overnight at 10°C. (C) We added 600 μL of WT, M8, M9, and buffer A in 1.5-mL clear tubes and stored them overnight at 4°C. (D) The samples were centrifuged at 21,600 ×*g* for 48 h at 4°C. The sample tubes in panels C and D were placed on yellow ferrules (from the 1/8" o.d. tubing connectors of GE Healthcare for AKTA systems) to allow them to stand on the desk.

The LLPS propensity of all mAbs at 50 mg/mL in low-ionic-strength buffer A is shown in Fig 3A. Single-charge deletion mutants (M1, M2, and M3) still underwent LLPS at 4°C however, they required more time to undergo LLPS than the WT. The WT underwent LLPS within 3 h of incubation at 4°C, whereas the appearances of mutants M1, M2, and M3 remained cloudy and homogeneous for several hours. These mutants ultimately underwent LLPS following overnight incubation at 4°C. LLPS was clearly mitigated in double- and triple-charge deletion mutants (M4, M5, M6, and M7), even after overnight incubation. M8 and M9 did not undergo clear phase separation at 4°C; however, their solutions were opaque, unlike those of M4–M7. In general, the opaque appearance could be due to both LLPS and fine precipitation.

To explain the opacity of M8 and M9 solutions, we conducted a larger-scale LLPS experiment with centrifugation. M8 and M9 solutions showed opacity after 24 h incubation at 4°C (Fig 3C). Subsequent centrifugation for 48 h at 4°C resulted in clear phase separation for M8 and M9, indicating that the opaque appearances of M8 and M9 solutions are due to LLPS (Fig 3D).

With temperature elevation from 4°C to 10°C, LLPS was maintained in the WT and mitigated in M1, M2, M3, M8, and M9. At 10°C, only M1, M2, and M3 solutions were opaque (Fig 3B), whereas M8 and M9 solutions were clear. Therefore, the LLPS propensity rank order was WT > M1 = M2 = M3 > M9 = M8 > M4 = M5 = M6 = M7. With regard to the lower phase, M8 and M9 solutions in the 1.5-mL tubes (Fig 3D) were clearer than M1, M2, and M3 solutions in PCR tubes (Fig 3A), probably because only M8 and M9 were subject to strong centrifugation for a long time.

The *B*_2_ values for these mAbs were determined using the AUC-SE method [28–30] (Table 1). A negative *B*_2_ value denotes attractive protein–protein interactions, whereas a positive value denotes mutual repulsion. The *B*_2_ values of all single-charge deletion mutants increased from that of the WT; however, their values were still negative. The double and triple-charge deletion mutants, which showed no LLPS, had positive *B*_2_ values. The triple-charge deletion mutant M7 had the highest positive *B*_2_ value of 3.6 among the tested mAbs.

**Fig 4:**
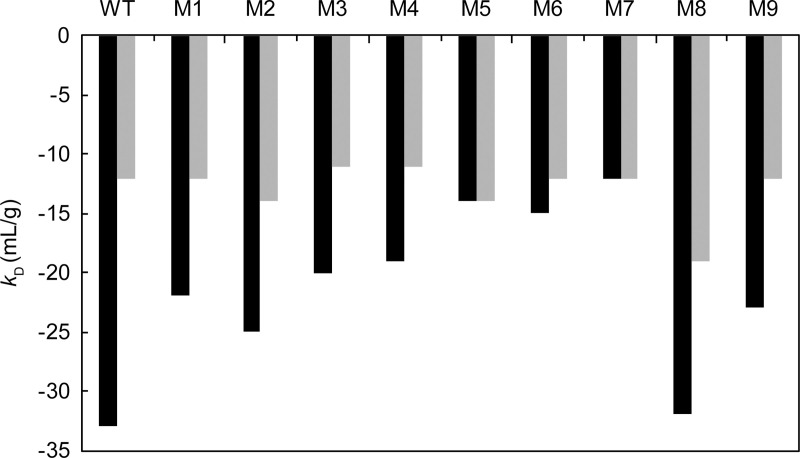
*k*_D_ values in the presence of 15 mM NaCl and 150 mM NaCl. Black bars show *k*_D_ values in buffer A containing 15 mM NaCl. Gray bars show *k*_D_ values in buffer B containing 150 mM NaCl.

The *k*_D_ value was determined by dynamic light scattering (Table 1) and was increased in proportion to the number of charge deletions. The *k*_D_ values were −33 ml/g for WT, −20 to −25 mL/g for the other single-charge deletion mutants, −14 to −10 mL/g for the double-charge deletion mutants, and −12 mL/g for the triple-charge deletion mutant. Adding 150 mM salt increased the *k*_D_ of the WT and mutants to a range of −11 to −19 mL/g (Fig 4). These results indicated that the deletion of negative charges effectively reduced attractive self-interactions and suggested that with regard to the WT, the electrostatic interaction could play a role as a a part of the soft interaction.

The relative solubility of the mAbs was evaluated by PEG precipitation. The value of *PEG*_*m*_ increased proportionally with the number of Ala-substituted acidic residues, which ranged from 5.62% to 9.13%. All mAbs showing LLPS propensity (WT, M1, M2, M3, M8, and M9) had *PEG*_m_ values of less than 8%.

All the negative surface charge mutations showed reduced viscosity compared with the WT. M5 showed the lowest viscosity of 8.19 mPa·s (S3 Fig).

## Discussion

### Relationships between the dipole moment, *B*_2_, and LLPS

**Fig 5:**
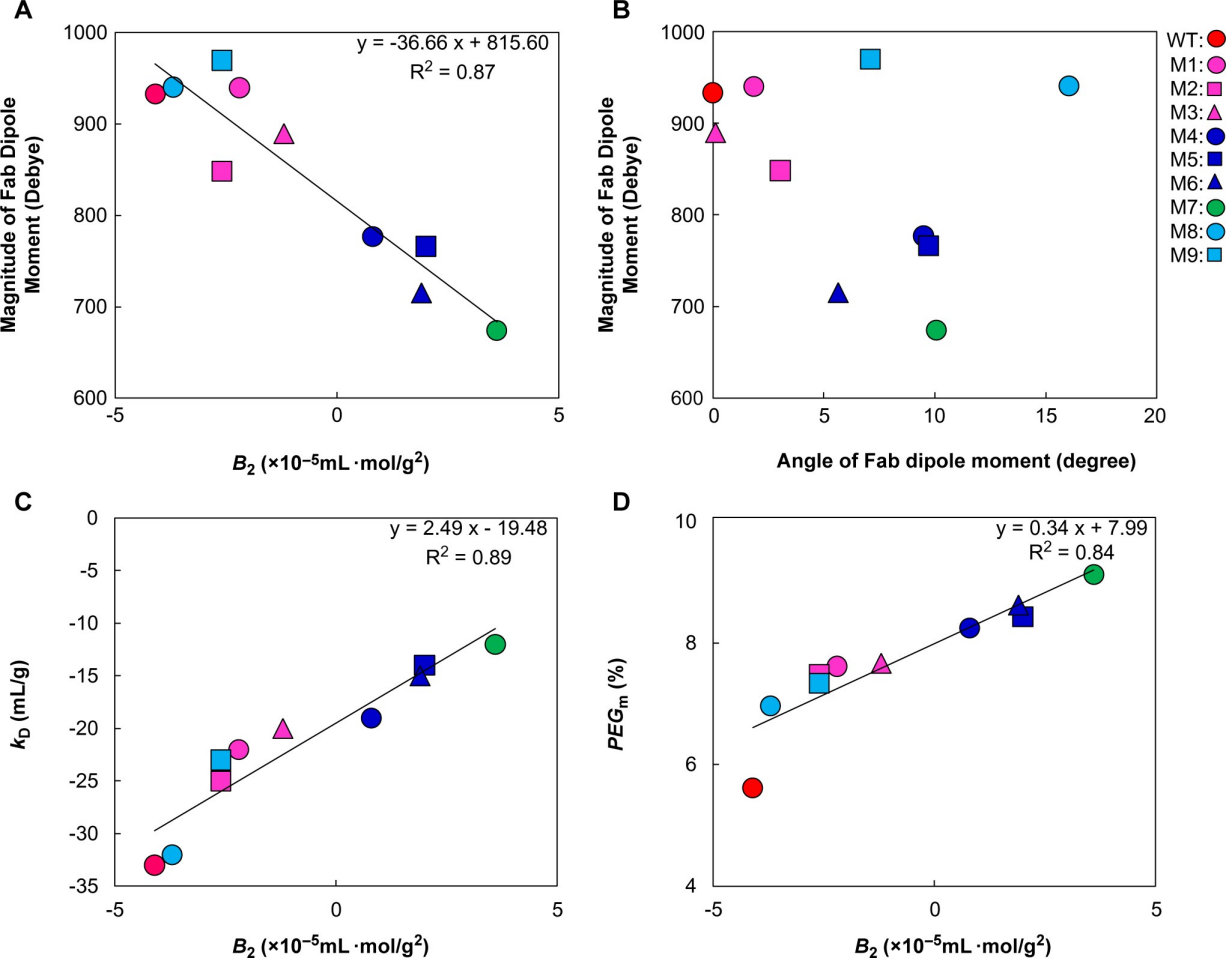
Correlations among various biophysical parameters of WT mAb1 and mutants in buffer A. (A) The magnitude of the Fab dipole moments was plotted against *B*_2_ values; (B) The angle between the WT dipole moment and each mutant dipole moment was plotted against the magnitude of the Fab dipole moment; (C) The *k*_D_ values were plotted against *B*_2_ values; (D) The *PEG*_m_ values were plotted against *B*_2_ values. Least-squares linear regression lines, correlation equations, and correlational coefficients are shown in (A), (C), and (D).

Our findings showed a strong correlation between *B*_2_ and the Fab dipole moment of single-, double-, and triple-charge deletion mutants (Fig 5A), indicating that the association can be described as being driven by a Fab dipole–dipole attraction. Kanai et al. [33] described the Fab–Fab interaction for protein–protein self-attractive interactions. Gentiluomo et al. [34] characterized the native reversible self-association of mAbs using Fab and Fc fragments and confirmed that self-associations such as hydrophobic and electrostatic interactions are driven by the Fab fragment. In addition, Du et al. [24] and Chow et al. [25] identified the amino acids responsible for the LLPS propensity of mAbs, located in the Fab region. In this study, we calculated Fab dipole moments to investigate the Fab–dipole-related association. In addition, limited numbers of entire IgG crystal structures are available, making the calculation of the entire IgG dipole moment difficult because the full-length model is only a single snapshot and cannot be interpreted as a representation of multiple hinge-angle forms. The dipole moment of the entire mAb differs in magnitude and direction from the Fab dipole moment. The computational approach we followed using a Fab model structure provided insight into a better design of LLPS mitigation mutants. However, further *in vitro* analysis, such as X-ray crystal structure analysis, is required to obtain evidence of Fab–Fab interaction mediating protein network formation of the mAb1 heavy phase.

The 10 mAbs were divided into two groups. The first group comprised four mAbs containing double and triple mutants that did not show LLPS propensity and had positive *B*_2_. The second group comprised the remaining six mAbs that showed LLPS propensity and had negative *B*_2_. Therefore, the sign of *B*_2_ could appear as an indicator of the LLPS propensity for mAb1. More precisely, *B*_2_ should be normalized to *B*_2_* while considering the excluded volume, as shown in Eq (2) [35]. Here, *B*_2ex_ indicates the contribution of the excluded volume to the virial coefficient. Negative *B*_2_* denotes protein–protein self-attractive interactions, while positive *B*_2_* denotes mutual repulsion. We are unaware of the actual contribution of *B*_2ex_ to the sign of *B*_2_*, but the sign of *B*_2_ appears to be well correlated with LLPS propensity in this study.

$$B_{2}^{*}=\frac{\left(B_{2}-B_{2ex}\right)}{B_{2ex}}$$ (2)

The LLPS propensity correlated with *B*_2_, and the *B*_2_ value had a linear relationship with the dipole moment. While LLPS propensity is a macroscopic phenomenon observed at high protein concentrations, *B*_2_ values are measured at relatively low protein concentrations. Accordingly, the dipole moment certainly contributes to *B*_2_, even at the high protein concentrations in this study. It is natural that not only the dipole moment but also other soft protein–protein interactions might play a role in determining *B*_2_. Saito et al. [36] also indicated a positive correlation between *B*_2_ values and viscosity in highly concentrated mAb solutions. They speculated that a similar force component, such as the dipole moment, could work not only at low concentration but also at high concentration. This supported the findings of our study.

### Significance of the dipole moment as an index for LLPS mitigation

The LLPS propensity correlated well with the magnitude of the Fab dipole moment, except for the outside-patch mutants M8 and M9. Despite exhibiting a Fab dipole moment of almost the same magnitude as the WT, the outside-patch M8 and M9 mutants did not undergo clear LLPS without centrifugation (Fig 3). The M8 and M9 LLPS propensity rank order could not be interpreted from the magnitude of the Fab dipole moment.

We further investigated the reason why M8 and M9 had lower LLPS propensities by plotting the angle between the WT dipole moment and each mutant dipole moment against the magnitude of the Fab dipole moment (Fig 5B). M8 and M9 had larger angles of the dipole moments than the other three single-charge deletion mutants (M1, M2, and M3). Our study indicated that the dipole moment could play a role in LLPS propensity. It is expected that the angle of Fab dipole moment affects the alignment of the molecule and protein–protein network formation in a crowded heavy phase. Therefore, the weak LLPS propensity of M8 and M9 could be explained by the angle to the WT Fab dipole moment. The results revealed that both the magnitude and the direction of the dipole moment could be essential for the LLPS propensity of mAb1, and the dipole moment could be the referential index for risk mitigation of the LLPS propensity.

Chow et al. [25] used X-ray crystal structure analysis to determine the amino acid residues of mutations that would mitigate the LLPS propensity. In the present study, we designed mutants based on tertiary structural models alone, without laborious *in vitro* experiments such as crystallization. We also calculated the Fab dipole of 10 mAbs from their primary structure within half a day. This *in silico* analytical approach could work in conjunction with high-throughput LLPS assessment in the course of drug candidate screening. In addition, the approach provides a reliable protocol for designing a mutant that does not show LLPS propensity in cases where electrostatic interaction is the dominant mechanism for LLPS. It reduces the number of candidate molecules required for *in vitro* LLPS assessment, thereby greatly improving operational efficiency.

### Relationships among various biophysical parameters other than the dipole moment

The *k*_D_ values strongly correlated with the *B*_2_ value (Fig 5C; squared correlation coefficient = 0.89). A *k*_D_ value of −20 mL/g was a threshold for LLPS where the *B*_2_ value indicated zero in this case study. Previous studies [28–30, 35–38] have also demonstrated a linear relationship between the *k*_D_ and *B*_2_ values. The investigations by Connolly et al. [28] indicated this relationship among eight different mAbs and corroborated our results.

All the negative surface charge mutations clearly reduced the viscosity of the WT. Moderate correlation between viscosity and the *B*_2_ value was shown with a squared correlation coefficient of 0.58 (S3 Fig). Previous studies assessing viscosity [19, 20] also suggested that the elimination of the uneven distribution of surface charges reduced viscosity. Because both LLPS and viscosity are a result of protein–protein self-attractive interactions, the two phenomena are correlated. LLPS is qualitatively correlated with *B*_2_. The viscosity of the WT and mutants is moderately correlated with *B*_*2*_, probably because of the nonlinear dependence of viscosity on the protein concentration. The viscosity of mAbs could be proportional to the exponential function of concentration [39].

The squared correlation coefficient between *B*_2_ and *PEG*_m_ was 0.84, indicating a strong correlation. The excluded volume theory predicts that PEG can trap H_2_O, thereby sterically excluding proteins from the solvent regions occupied by PEG and causing a phase transition of proteins [40]. Here, the electrostatic interaction could be the major interaction in a low-ionic-strength buffer such as buffer A. The electrostatic interaction also reflected interaction parameters such as *B*_2_ for our charge mutants in buffer A. Thus, the surface charge mutants showed correlations of *B*_2_ with *PEG*_m_. (Fig 5D).

## Conclusions

We demonstrated the mitigation of LLPS by deletion of negative charge patches from the surface of the mAb1 Fv region. LLPS propensity was eliminated for the mutants with positive *B*_2_ values, with reducing theoretical Fab dipole moments. Therefore, the sign of *B*_2_ may be useful for predicting LLPS propensity. Protein–protein soft interactions are complex; however, both the magnitude and the direction of the dipole moment could be part of the essential contributors to the LLPS propensity where the dominant mechanism for LLPS is electrostatic interaction. Further studies are required to apply these findings to other mAbs. The insights obtained in this study could open up new avenues for the design and selection of well-behaved therapeutic mAbs.

## Supporting information

S1 Fig Dipole moment and electrostatic potential surfaces of Fabs for WT and mutants mAbs. (A) WT, (B) M1, (C) M2, (D) M3, (E) M4, (F) M5, (G) M6, (H) M7, (I) M8, and (J) M9. Red and blue contours indicate −1 and +1 *k*_B_*T*/*e* isosurface potentials. (K) Corresponding transparent isosurface potential depicted next to each model to highlight the dipole moment. The dipole moment is illustrated in both transparent and dense isosurface potential maps. The length does not show the dipole moment magnitude but indicates the scalar amount of the magnitude projected on a plane parallel to the paper.
(TIF)

S2 Fig DSC thermograms of WT mAb1 and all mutants. (TIF)

S3 Fig Correlation between *B_2_* values and viscosity. The correlation equation was determined by least-squares linear regression analysis.
(TIF)

S1 Table Dipole moment of the Fab models. ^a^Dipole: X, Dipole: Y, and Dipole: Z are the x-, y-, and z-components of the Fab dipole moment. The angle between the WT dipole moment and each mutant dipole moment was calculated from the Dipole X, Dipole Y, Dipole Z values.
(DOCX)
