# Supplementary figures and images for: Mitigation of liquid–liquid phase separation of a monoclonal antibody by mutations of negative charges on the Fab surface
Source: PLoS One. 2020 Oct 30;15(10):e0240673. doi: 10.1371/journal.pone.0240673 (PMC7598502; doi:10.1371/journal.pone.0240673)

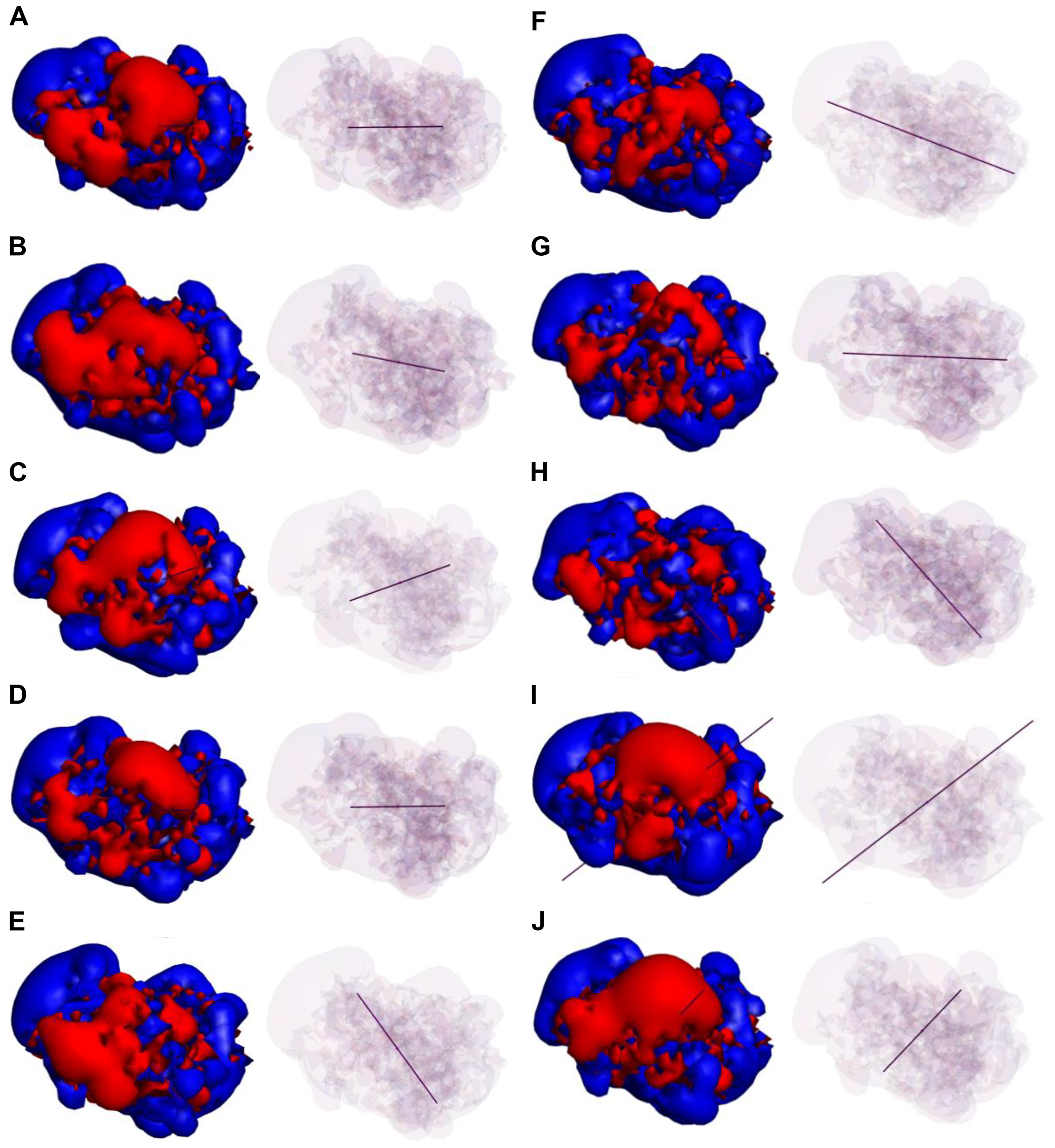

Supplement: S1 Fig — (A) WT, (B) M1, (C) M2, (D) M3, (E) M4, (F) M5, (G) M6, (H) M7, (I) M8, and (J) M9. Red and blue contours indicate −1 and +1 kBT/e isosurface potentials. (K) Corresponding transparent isosurface potential depicted next to each model to highlight the dipole moment. The dipole moment is illustrated in both transparent and dense isosurface potential maps. The length does not show the dipole moment magnitude but indicates the scalar amount of the magnitude projected on a plane parallel to the paper. (TIF) [file pone.0240673.s001.tif]

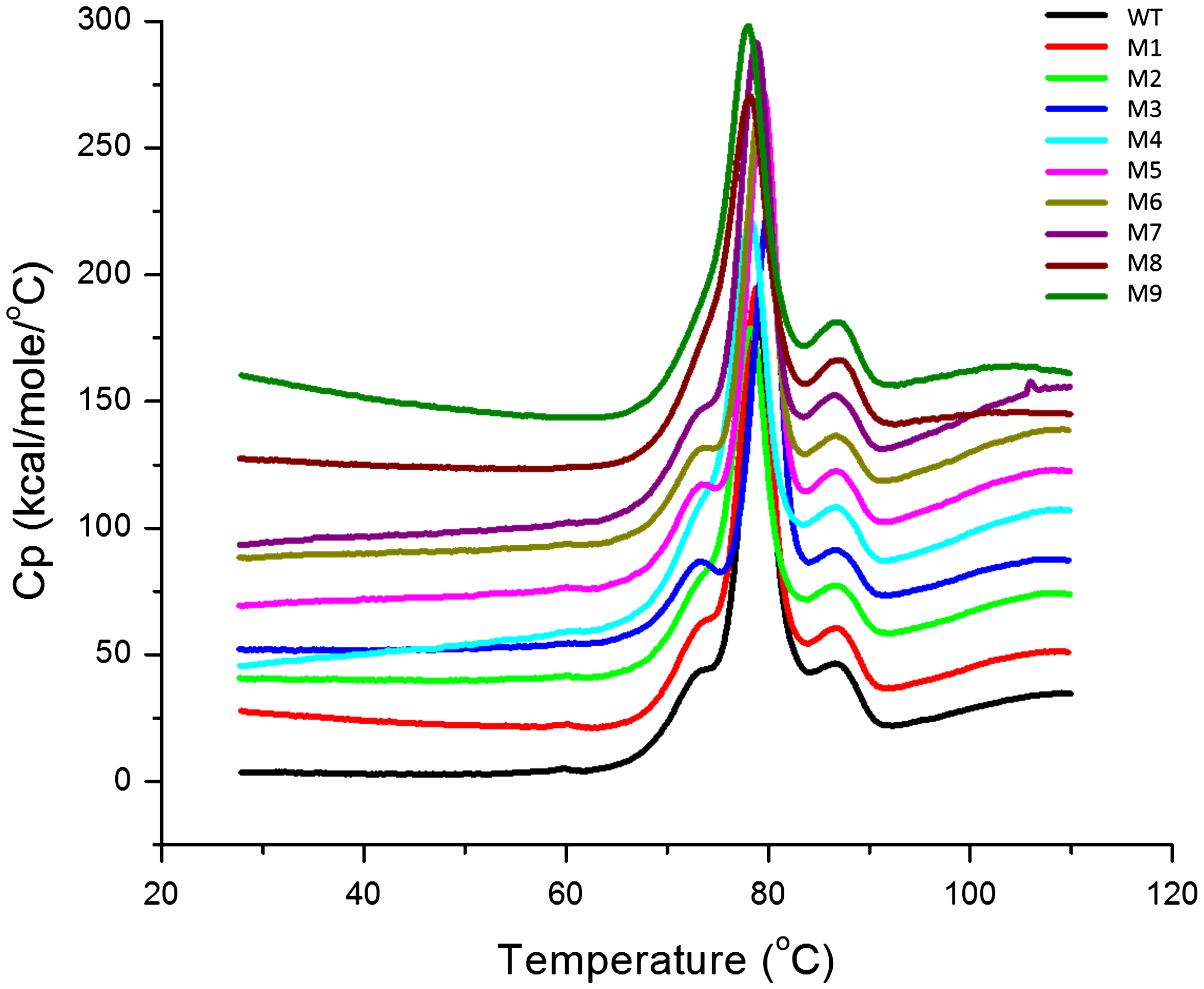

Supplement: S2 Fig — (TIF) [file pone.0240673.s002.tif]

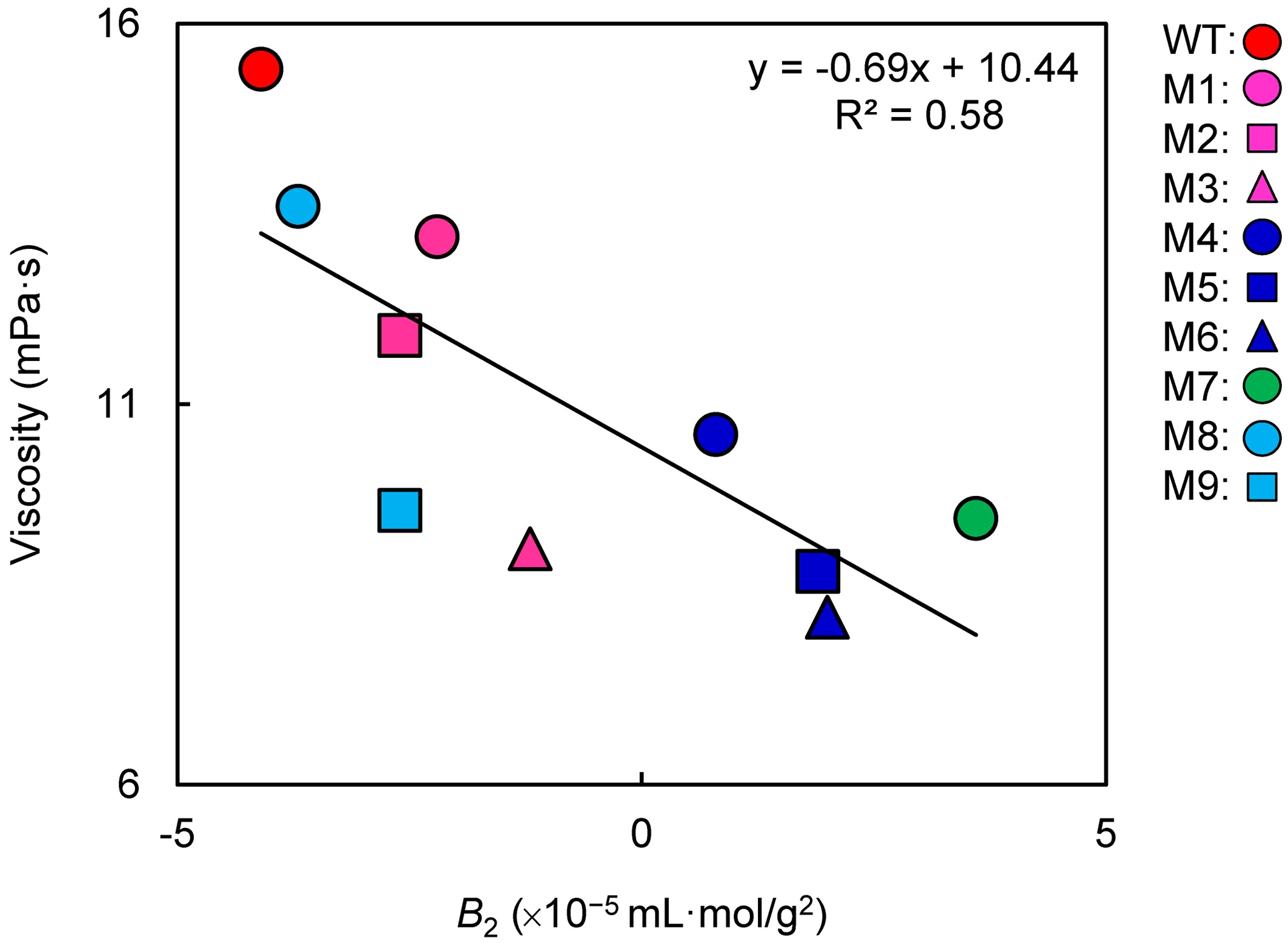

Supplement: S3 Fig — The correlation equation was determined by least-squares linear regression analysis. (TIF) [file pone.0240673.s003.tif]
