# Supplementary material for: Mitigation of liquid–liquid phase separation of a monoclonal antibody by mutations of negative charges on the Fab surface
Source: PLoS One. 2020 Oct 30;15(10):e0240673. doi: 10.1371/journal.pone.0240673 (PMC7598502; doi:10.1371/journal.pone.0240673)
Supplement: S1 Table — aDipole: X, Dipole: Y, and Dipole: Z are the x-, y-, and z-components of the Fab dipole moment. The angle between the WT dipole moment and each mutant dipole moment was calculated from the Dipole X, Dipole Y, Dipole Z values. (DOCX) [file pone.0240673.s004.docx]

**S1 Table. Dipole moment of the Fab models**

| Name of mAb1 | Dipole: X^a^ | Dipole: Y^a^ | Dipole: Z^a^ | Magnitude of dipole moment (debye) | Angle between the wild type (WT) dipole moment and each mutant dipole moment (°) |
| --- | --- | --- | --- | --- | --- |
| WT | 185.9 | −793.4 | −454.4 | 933.0 | - |
| M1 | 194.5 | −784.2 | −479.7 | 939.7 | 1.68 |
| M2 | 159.6 | −743.6 | −375.4 | 848.2 | 3.02 |
| M3 | 178.6 | −754.0 | −431.5 | 886.9 | 0.13 |
| M4 | 178.1 | −584.7 | −479.6 | 776.9 | 9.50 |
| M5 | 56.7 | −615.3 | −442.7 | 760.1 | 9.30 |
| M6 | 73.9 | −611.0 | −364.2 | 715.1 | 5.65 |
| M7 | 130.1 | −506.2 | −425.7 | 674.1 | 10.07 |
| M8 | 20.6 | −897.5 | −280.6 | 940.6 | 16.04 |
| M9 | 209.1 | −874.1 | −364.3 | 969.8 | 7.08 |
